# Bimetallic Implanted Plasmonic Photoanodes for TiO_2_ Sensitized Third Generation Solar Cells

**DOI:** 10.1038/s41598-020-64653-6

**Published:** 2020-05-06

**Authors:** Navdeep Kaur, Viplove Bhullar, Davinder Paul Singh, Aman Mahajan

**Affiliations:** Department of Physics, Guru Nanak Dev University, Amritsar, 143 005 India

**Keywords:** Energy science and technology, Materials science, Physics

## Abstract

An auspicious way to enhance the power conversion efficiency (PCE) of third generation sensitized solar cells is to improve the light harvesting ability of TiO_2_ sensitizer and inhibition of back recombination reactions. In the present work, we have simultaneously comprehended both the factors using stable bimetallic Au and Ag metal nanoparticles (Mnps) embedded in TiO_2_ with ion implantation technique at lower fluence range; and explored them in third generation dye sensitized solar cells (DSSCs). The best performing Au-Ag implanted DSSC (Fluence- 6 × 10^15^ ions cm^−2^) revealed 87.97% enhancement in its PCE relative to unimplanted DSSC; due to plasmon induced optical and electrical effects of Mnps. Here, optimized bimetallic Au-Ag Mnps embedded in TiO_2_ improves light harvesting of N719 dye; due to the well matched localized surface plasmon resonance (LSPR) absorption band of Au and Ag with low and high energy absorption bands of N719 dye molecules, respectively. Furthermore, Au and Ag acts as charge separation centers in TiO_2_ that inhibit the recombination reactions occurring at photoanode/electrolyte interface *via* prolonging photo-generated electron lifetime; resulting in efficient inter-facial charge transportation in DSSCs.

## Introduction

In last decade, third generation sensitized solar cells have achieved tremendous consideration out of authoritative traditional silicon based photovoltaic technology in tandem cell configuration; owing to their low manufacturing cost, non-toxic nature and undeniable higher theoretical limit of power conversion efficiencies (PCE)^1–5^. Sensitizer plays a key role in third generation sensitized solar cells; thus should exhibit phenomenal properties like high chemical and thermal stability, high photo-catalytic activity, low-cost, high redox ability, strong absorption coefficient, biocompatibility, high specific surface area, non-toxicity, and recyclability^6^. TiO_2_, an extrinsic n-type semiconductor, fulfills all the requirements and hence widely studied as sensitizer in third generation photovoltaic devices constituting quantum dot sensitized solar cells, dye sensitized solar cells (DSSCs), and pervoskite sensitized solar cells^7^. However, TiO_2_ appears to undergo some limitations such as random charge transportation occurring due to trapping and detrapping of photo-generated electrons in its trap levels resulting in recombination losses and light absorption confined only in UV region of solar spectrum; hence requires some modifications which can be done *via* incorporating different shape, size and dimensions of TiO_2_, metal oxides, metal nanoparticles (Mnps), hybridized carbon materials, and coupling with semi-conducting materials^8–10^. Among them, inclusion of Mnps such as gold (Au) and silver (Ag) induce plasmonic optical and electrical effects in TiO_2_, due to their unique localized surface plasmon resonance (LSPR) property; which effectively strengthens their light absorption and charge transportation ability and ultimately improves the PCE of sensitized third generation solar cells^11–13^.

Recently, we observed that the single crystalline spherical shaped Ag nps incorporated in TiO_2_ *via* chemical reduction method, effectively improves the absorption cross-section of dye sensitizer^14^. Moreover, the inclusion of different single crystalline anisotropic shapes of Ag nps in TiO_2_ results in improved light harvesting ability of TiO_2_; as they exhibit multiple LSPR bands in the broadened region of solar spectrum ranging from 380–900 nm^15^. Although, bare Mnps significantly improves the light harvesting ability of TiO_2_ *via* enhancing the absorption cross-section of dye sensitizer; they become unstable coming in contact with liquid based electrolytes and gets corroded.

Xu *et al*. demonstrated the utilization of Au-Ag alloy popcorn shaped core-shell nanoparticles in TiO_2_ to broaden the light absorption range *via* using efficient excitation of LSPR modes on the popcorn nanoparticles^16^. However, core-shell structures improved the stability of Mnps in liquid electrolytes, but suffered from their unpronounced LSPR effect on light harvesting ability of TiO_2_.

Furthermore, ion implantation have been observed to be practically quite effective technique to embed Mnps in TiO_2_; which helped in resolving both the instability and unpronounced LSPR issues of bare and core-shell structures, respectively^17,18^. Recently, we have explored the ion implantation method to implant Au Mnps in TiO_2_ and employ them as photoanodes in third generation DSSCs. The optimized Au implanting fluence provides balancing effect of LSPR of Au and effective adsorption area of TiO_2_ for N719 dye in DSSCs; and showed an enhancement of 44.7% in its PCE relative to unimplanted TiO_2_ based DSSCs^19^. Similarly, Ag Mnps implantation in TiO_2_ showed 65.3% enhancement in PCE of DSSC^20^. However, their increment is not a match for its economical utilization; since efficient light harvesting in Au as well as Ag implanted DSSCs is only around single LSPR absorption wavelength; resulting in unsatisfactory PCEs.

In order to further enhance the light harvesting ability of TiO_2_ within the whole visible region of solar spectrum, Kim *et al*. demonstrated the efficient energy matching between the absorption bands of N719 dye and Au and Ag Mnps; resulting in relatively enhanced PCE of double layered plasmonic DSSCs by 19.12%^21^. Wang *et al*. introduced the plasmonic cooperation of Au and Ag Mnps in TiO_2_ photoanodes of DSSCs using chemical reduction method and achieved 20.8% PCE enhancement; by exploiting the strong plasmonic cooperation effects due to the complementary light absorption of both the Au and Ag Mnps *via* their respective LSPR absorptions at ~550 and 400 nm^22^. Yun *et al*. demonstrated the incorporation of core shell Au@Ag Mnps in TiO_2_ hollow spheres leading to synergistic effects of improved light harvesting and widening of absorption band, appearing due to effective light scattering effects of core-shells; which raised the PCE by 25%^23^. Dong *et al*. proclaimed 40% increment in PCE of DSSCs comprising Ag-encapsulated Au nanorods prompting enhanced light harvesting and efficient one dimensional charge transportation^24^. Al-Awazi *et al*. showed the optimal concentration of Au-Ag alloys (4:1) introduced into TiO_2_ *via* pulsed laser ablation, emanating PCE by 52.1%; arising from the broader optical absorption of dye molecules using plasmonic effects of Au and Ag Mnps generating larger number of photo-generated charge carriers^25^. A seed mediated growth of Ag shells on Au Mnps incorporated in TiO_2_ photoanodes have been investigated by Salimi and his co-workers, and noted 125% enhancement in PCE of DSSCs due to their prominent light absorption as well as enhanced inter-facial charge transportation through Mnps minimizing the charge recombination processes^26^. Till now, maximum 230% enhancement in PCE of DSSCs, having modified TiO_2_ photoanodes with Au-Ag nanocomposites, have been achieved by Lim *et al*. that originates from LSPR synergistic interactions between Au and Ag Mnps resulting in the improved light harvesting; and efficient charge separation and transportation processes^27^.

Above reported literature mentioned the simultaneous inclusion of Au and Ag Mnps in photoanodes of DSSCs using chemical methods, and their PCEs enhancement is not at par with the theoretical maximum value; resulting from the uncontrolled growth and concentration of Mnps which leads to their non-linear conglomeration in TiO_2_; hence deteriorating their long term stability and sustainability. In this direction, we have investigated the use of ion implantation technique to embed Au and Ag Mnps inside TiO_2_ semiconductor of DSSCs; resulting in efficient enhancement in the PCE of highly stable plasmonic DSSCs *via* extending the region of light harvesting within UV-Vis-NIR, by utilizing LSPR absorptions of Au and Ag Mnps as well as improving the charge transfer processes by reducing recombination rate of photo-generated charge carriers, owing to their charge storage ability.

## Results and Discussion

### Concept visualization

**Figure 1:**
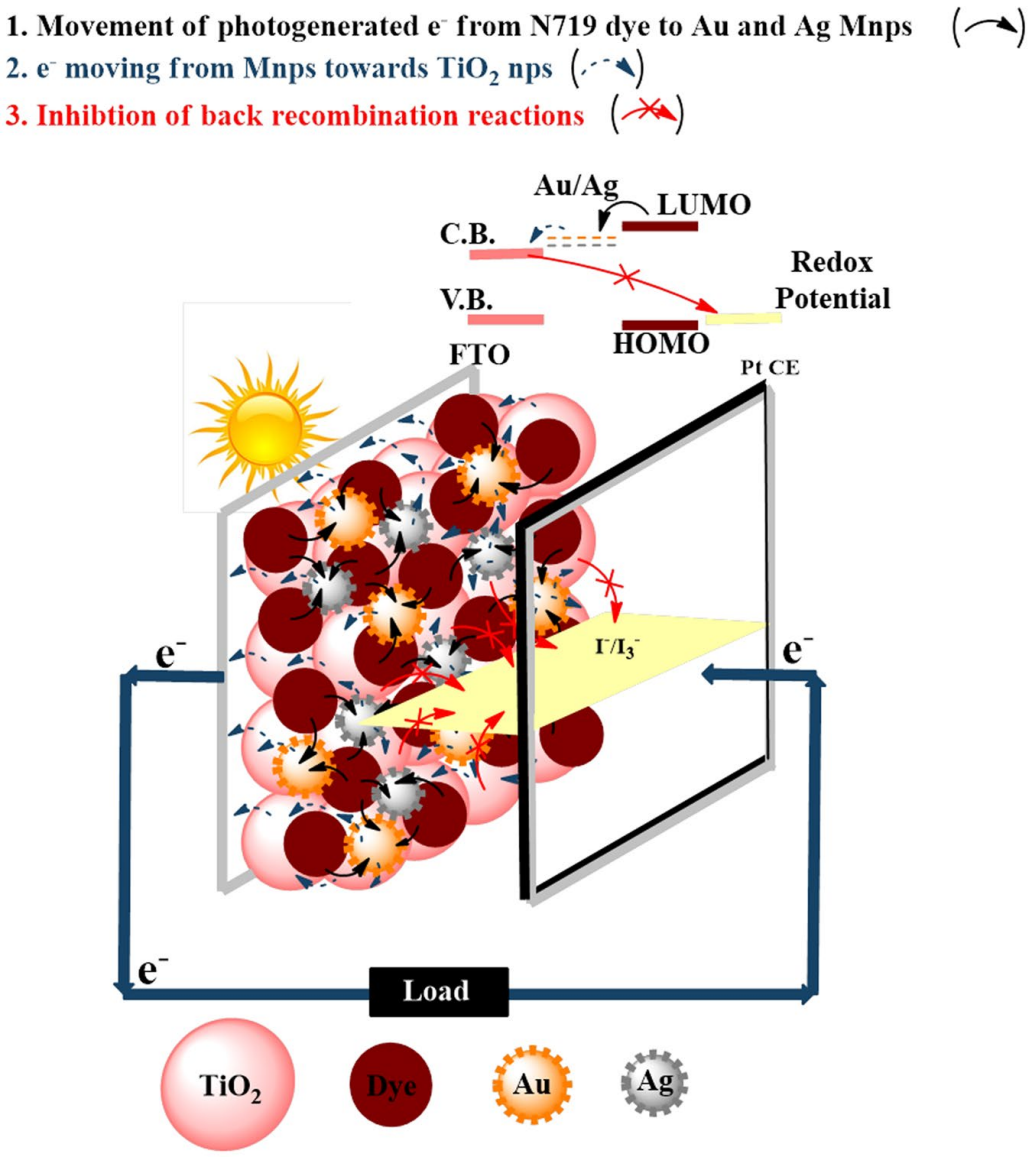
Proposed concept visualization. Schematic diagram of bimetallic Au and Ag implanted plasmonic DSSC.

Bimetallic Au and Ag implantation in TiO_2_ will significantly improve its light harvesting ability and inhibit back recombination reactions, resulting in better electron transportation; that positively influences the photovoltaic performance of DSSCs. Figure 1 represents the schematics of electron transportation processes occurring in DSSCs using bimetallic Au-Ag implanted TiO_2_ photoanodes. When light falls on the photoanode of DSSCs, enormous photo-generated electrons are produced from excited N719 dye molecules, especially from the ones present in the vicinity of Au and Ag Mnps. Rapid accumulation of photo-generated electrons on Au and Ag Mnps takes place due to their charge storage ability (Fig. 1(1)); which in turn induces an upward shift in the Fermi energy level (E_F_) of photoanode; resulting in efficient transportation of accumulated photo-generated electrons to the conduction band (C.B.) of TiO_2_ (Fig. 1(2))^28^. Moreover, the trap levels of TiO_2_ are filled by the electrons generated from the plasmonically excited Au and Ag Mnps, which reduces the random charge distribution of photo-generated electrons in TiO_2_; hence inhibits the recombination reactions occurring at photoanode/ electrolyte interface (Fig. 1(3))^29^. Further, the photo-generated electrons from the C.B. of TiO_2_ get transported to the external circuit *via* TiO_2_ compact layer deposited FTO and are collected at Pt CE^30^. Thus, Au and Ag Mnps in TiO_2_ would efficiently enhance the charge transportation processes throughout DSSC *via* elongating electron lifetime as well as transport path length. The above mentioned concept exhibiting higher light harvesting ability as well as better charge transportation processes in bimetallic Au and Ag implanted photoanode based DSSCs has been fully supported by the underlying investigations.

### Structural and Optical characterization of photoanodes

**Figure 2:**
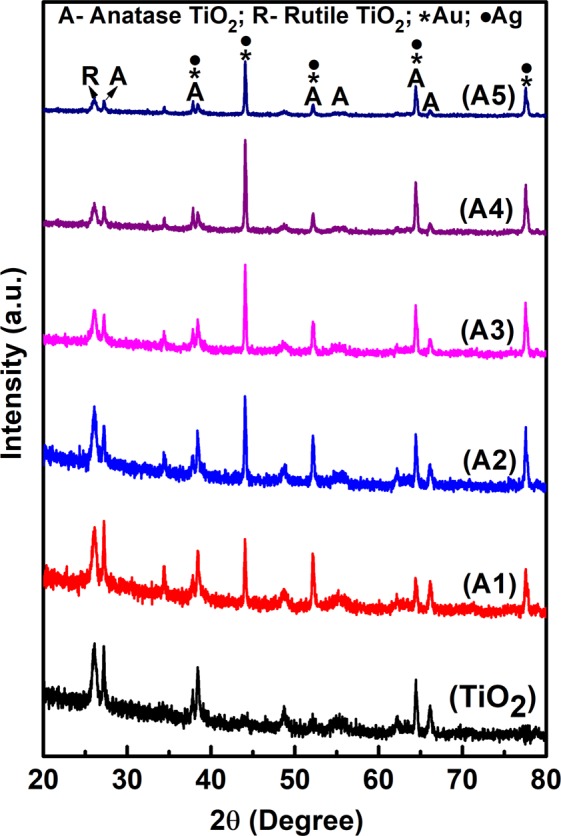
Crystal structure. X-ray diffraction patterns of unimplanted and Au-Ag implanted TiO_2_ at varying fluence (A1–A5).

XRD diffraction patterns of unimplanted and Au-Ag implanted TiO_2_ on FTO substrates are depicted in Fig. 2. TiO_2_ exists in both the anatase (A) and rutile (R) phase with peaks appearing at 26.08°, 27.22°, 38.42°, 48.66°, 54.96°, 64.48°, and 66.18° Bragg’s angle relating to (110)-R, (110)-A, (004)-A, (200)-A, (211)-A, (116)-A, and (204)-A crystal planes, respectively (JCPDS Card No. 21–1272 and 04-0551). In case of Au-Ag implantation in TiO_2_, two new crystal planes (200) and (311) at 44.04° and 77.58° Bragg’s angle, respectively appeared along with slight increase in the intensity of (004), (211), and (116) plane of TiO_2_; which is due to the overlapping of Au, Ag and TiO_2_ crystal planes; as they exhibit very similar lattice constants that further confirms their metallic face centered cubic crystal structure (JCPDS Card No. 04-0784 for Au and 04-0783 for Ag). The peak intensities with overlapped Au, Ag and TiO_2_ crystal planes enhances initially with increasing Au-Ag implantation fluence, that results in the formation of large number of metal nanoparticles (Mnps); but then suddenly decreases which is due to the suppression of TiO_2_ planes as they get covered with Au and Ag Mnps as well as thin TiO_2_ melt. Thus, the crystallinity of Au-Ag implanted TiO_2_ increases particularly along (200) and (311) crystal planes; leading to better charge carrier mobility which is necessity for improved DSSCs.

**Figure 3:**
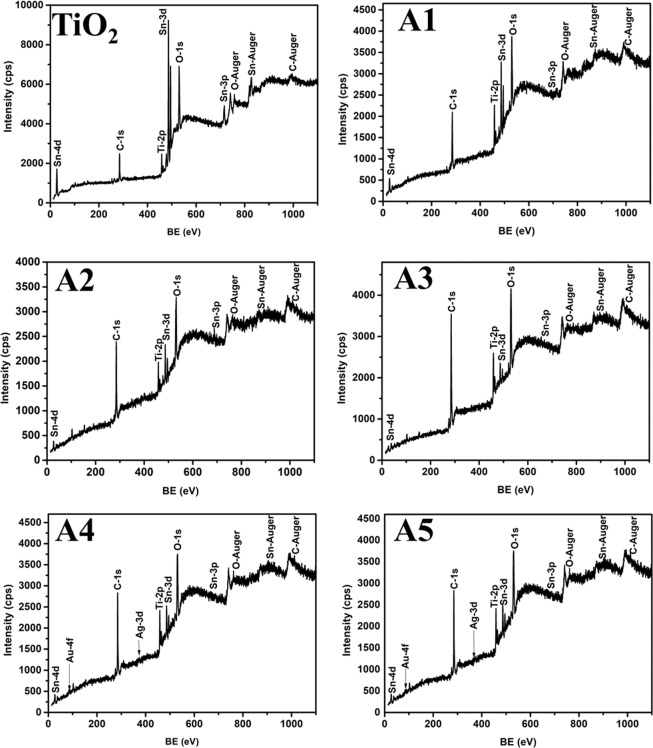
Composition analysis. XPS survey spectra of unimplanted and Au-Ag implanted TiO_2_ at varying fluence (A1–A5).

**Figure 4:**
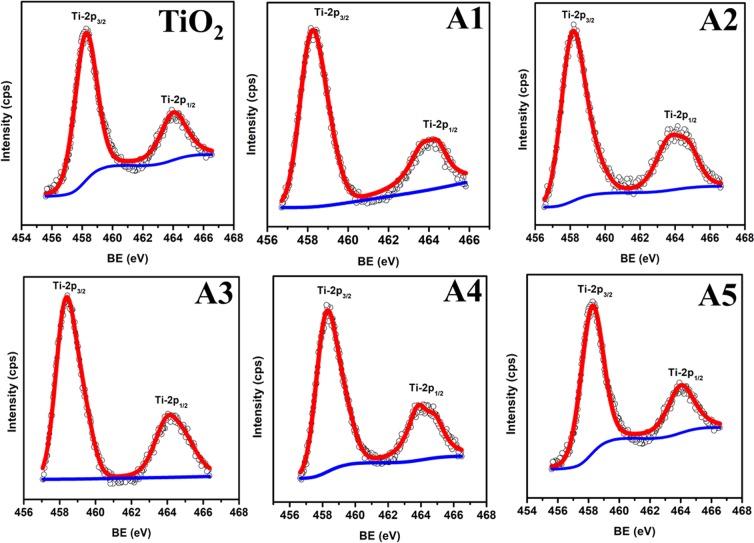
Titanium composition. High resolution Ti-2p XPS spectra of unimplanted and Au-Ag implanted TiO_2_ at varying fluence (A1–A5).

**Figure 5:**
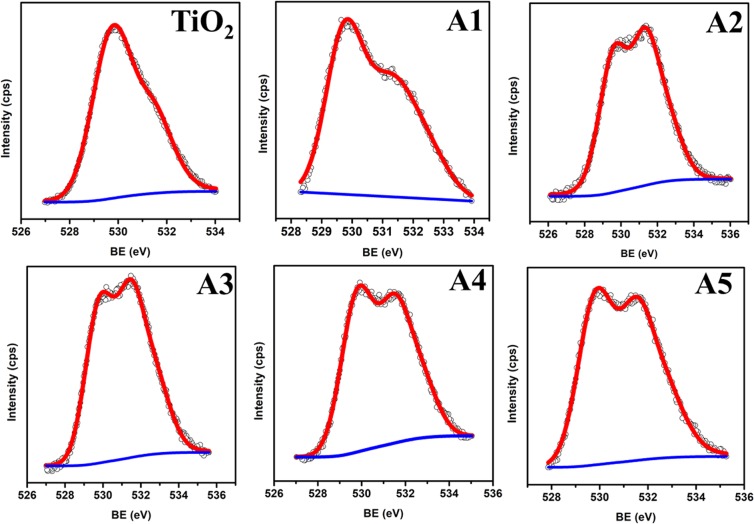
Oxygen composition. High resolution O-1s XPS spectra of unimplanted and Au-Ag implanted TiO_2_ at varying fluence (A1–A5).

The elemental composition as well as chemical state of unimplanted and Au-Ag implanted TiO_2_ at varying fluence are analyzed from the XPS survey spectra demonstrated in Fig. 3; which shows four peaks of Ti-2p, O-1s, Au-4f and Ag-3d elements. The high resolution Ti-2p spectra of TiO_2_, present in all the samples (Fig. 4); exhibited two peaks centered at binding energies 458.3 and 464.3 eV corresponding to Ti-2P_3/2_ and Ti-2P_1/2_, respectively; which indicates the presence of Ti^4+^ oxidation state^27^. O-1s peak observed at binding energy 529.9 eV corresponds to the lattice oxygen atoms (O^2−^) in the TiO_2_; present in the form of oxides, −COO^−^ and >C=O in each sample (Fig. 5). No significant change in the binding energies of Ti-2P_3/2_, Ti-2P_1/2_ and O-1s is observed in Au-Ag implanted TiO_2_, which is a clear manifestation of unaffected nano-crystalline structure of TiO_2_.

**Figure 6:**
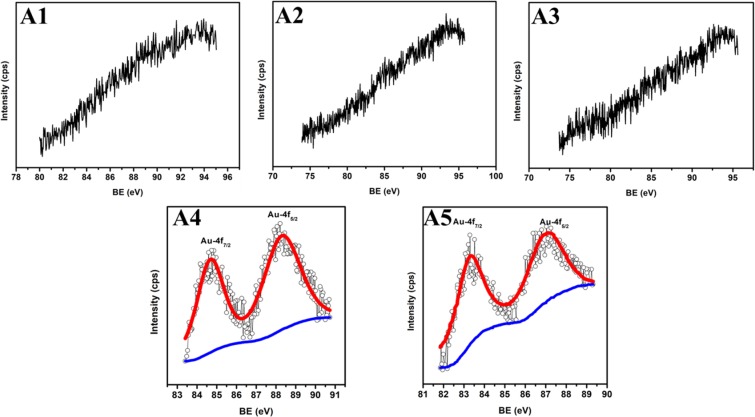
Au composition. High resolution Au-4f XPS spectra of Au-Ag implanted TiO_2_ at varying fluence (A1–A5).

**Figure 7:**
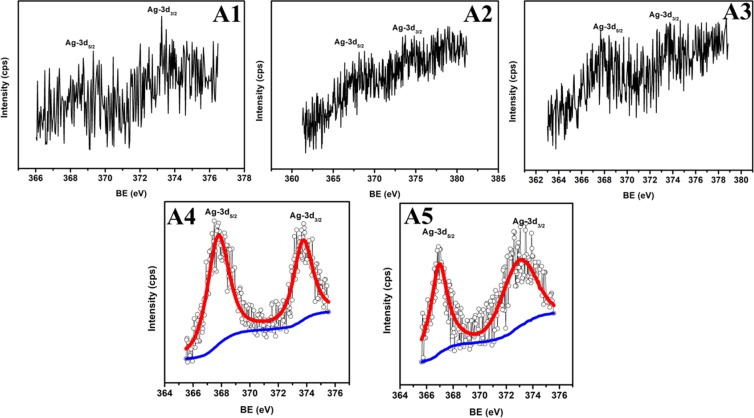
Ag composition. High resolution Ag-3d XPS spectra of Au-Ag implanted TiO_2_ at varying fluence (A1–A5).

After the implantation of Au-Ag in TiO_2_; very weak Au and Ag peaks present in Au 4f_7/2_, Au 4f_5/2_ and Ag-3d_5/2_, Ag-3d_3/2_ states appeared at binding energies 83.3, 87.0 eV (Fig. 6) and 367.0, 373.0 eV (Fig. 7), respectively; whose intensity increases with increasing fluence and becomes measurable at A4 and A5 samples. The difference between two states of Au (~3.7 eV) and Ag (~6.0 eV) clearly suggests their presence in zero valent metallic state *i.e*. Au^0^ and Ag^0^ ^27^. Moreover, the binding energies of Au-4f_5/2_ and Ag-3d_5/2_ are slightly smaller than their metallic state; which arises due to the electron transfer processes taking place from TiO_2_ to Au and Ag Mnps. The immeasurable Ag and Au atomic %age in A1, A2 and A3 samples indicates Au and Ag content lesser than the 0.5 atomic %age; which is confirmed through decreasing Ti element content. The atomic %age of Ti-2P_3/2_, Ti-2P_1/2_, O-1s, Au-4f_5/2_, Au-4f_7/2_, Ag-3d_5/2_ and Ag-3d_3/2_ elements are summarized in Table 1; showing an increased atomic %age of Ag and Au as well as decreased content of Ti with increased fluence in TiO_2_.

**Table 1:** Elemental composition.

Sample	Atomic %age of Ti	Atomic %age of O	Atomic %age of Au	Atomic %age of Ag
TiO_2_	44.84	55.15	—	—
A1	44.84	55.15	—	—
A2	41.90	58.10	—	—
A3	34.67	65.32	—	—
A4	27.35	39.38	19.68	13.7
A5	6.92	66.87	10.43	15.7

Atomic %age composition of Ti, O, Au and Ag elements present in unimplanted and Au-Ag implanted TiO_2_.

**Figure 8:**
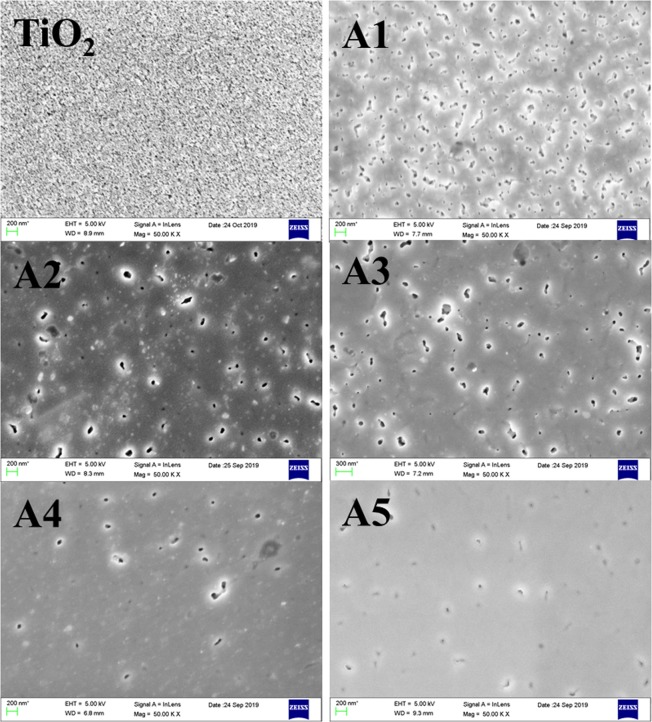
Morphology of TiO_2_. FESEM images of unimplanted and Au-Ag implanted TiO_2_ at varying fluence (A1–A5).

The FESEM images demonstrating the existence of Au and Ag Mnps embedded in TiO_2_ *via* modifying their surface morphology are depicted in Fig. 8. TiO_2_ surface depicts unequal as well as inter-connected nano-pores morphology; which gets flattened with Au-Ag implantation and the flattening increases with increased fluence; along with the increasing pore size distribution^31^. The uniform embedment of Au-Ag Mnps over flattened surface has been observed at Au-Ag implanted TiO_2_ and their number increases with increasing fluence.

**Figure 9:**
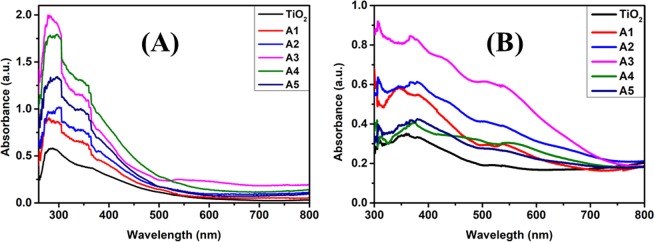
Absorption spectra. UV-Vis absorption spectra of **(A)** without and **(B)** with N719 dye loaded unimplanted and Au-Ag implanted TiO_2_ photoanodes at varying fluence (A1–A5).

The UV-visible absorption spectra of TiO_2_ and Au-Ag embedded TiO_2_ at different fluence are depicted in Fig. 9(A). TiO_2_ represents prominent absorption around 310 nm arising due to their intrinsic electronic excitation; with negligible absorption in the visible region of solar spectrum. The range of absorption has been observed to be extended to the visible light region with the implantation of Au and Ag Mnps in TiO_2_; attributable to the characteristic LSPR absorption peak of Au and Ag lying around 530 nm and 400 nm, respectively stimulated by optical excitation. Also, the LSPR phenomenon of Au and Ag Mnps enhances the absorbance of TiO_2_ in UV region. The absorbance peak shows a continuous increase with increased fluence up to A3; then a sudden decrement is observed along with the sharp decrease in TiO_2_ absorbance around 310 nm. It is related to the excessive flattening of TiO_2_ surface that reduces its absorption cross-sectional area; which is in consonance with FESEM images.

Similar trend has been observed in the absorbance spectra of N719 dye loaded TiO_2_, A1, A2, A3, A4 and A5 photoanodes and is shown in Fig. 9(B). After dye loading, TiO_2_ photoanode exhibited strong absorption peaks centered at 400 and 530 nm; which corresponds to the metal to ligand charge transfer interactions within N719 dye molecules. Higher absorbance around 400 nm is due to the merged absorption peaks of TiO_2_ as well as dye molecules. Broad absorption region extending from UV to NIR light region (300–750 nm) has been observed with the Au-Ag implantation; which is attributed to the synergistic effects of LSPR peaks at 400 and 530 nm associated with Ag and Au Mnps, respectively; that efficiently interacts with dipole moment of dye molecules within a certain minimal spatial range.

**Figure 10:**
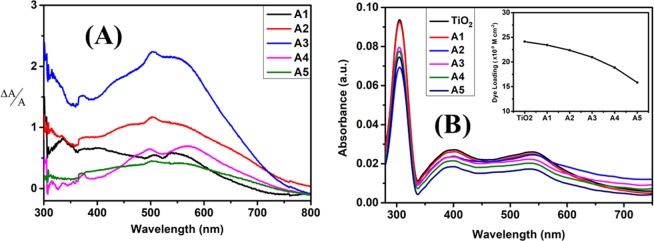
Relative absorbance change and dye deloading spectra. (**A**) Relative absorbance change in the Au-Ag implanted TiO_2_ photoanodes w.r.t. unimplanted TiO_2_; and **(B)** UV-Vis absorption spectra of N719 dye deloaded from different photoanodes (inset – represents the variation of amount of dye loading at different photoanodes).

The relative change in the absorbance of different Au-Ag implantation fluence based TiO_2_ photoanodes with respect to unimplanted TiO_2_ (Fig. 10(A)) for understanding their effective contribution in absorption enhancement in the entire wavelength range; has been estimated from Eq. (1) ^25^;

1 $$\frac{\Delta A}{A}=\frac{{A}_{Ti{O}_{2},N719,Au/AgMnps}(\lambda )-{A}_{Ti{O}_{2},N719}(\lambda )}{{A}_{Ti{O}_{2},N719}(\lambda )}$$

where, ${A}_{Ti{O}_{2},N719,Au/AgMnps}(\lambda )$ is the absorbance of dye loaded Au-Ag implanted TiO_2_ photoanodes and ${A}_{Ti{O}_{2},N719}(\lambda )$ represents the absorbance of unimplanted dye loaded TiO_2_ photoanodes. It confirmed the continuous enhancement in the relative absorbance up to A3 photoanode, which reflects the sole contribution of Au-Ag Mnps embedment in improving the light harvesting ability of TiO_2_ photoanodes. Although Au and Ag synergistically affect optical absorption of TiO_2_ photoanodes, the parallel decrease in the amount of dye loading starts to dominate at higher fluence; that leads to smaller relative absorbance change. Figure 10(B) depicts the dye desorption spectra of all the prepared photoanodes which shows absorbance peak around 400 and 530 nm related to N719 dye. The amount of dye loaded on the photoanodes can be estimated from Eq. (2) ^20^;

2 $${\rm{Dye}}\,{\rm{Loading}}({\rm{mole}}.{{\rm{cm}}}^{-2})=\frac{[{\rm{Dye}}\,{\rm{Concentration}}({\rm{in}}\,{\rm{M}})\times {\rm{Volume}}\,({\rm{ml}})]}{{\rm{Electrode}}\,{\rm{Area}}\,({{\rm{cm}}}^{2})}$$

where, dye concentration is calculated using Beer Lambert’s law at absorbance value around 535 nm, 10 ml volume is taken and effective area is 0.25 cm^2^. The variation of dye loading for TiO_2_, A1, A2, A3, A4 and A5 photoanodes, respectively shown in the inset of Fig. 10(B); suggests that at lower fluence, the dye loading is almost similar but at A4 and A5 fluence it is considerably small; hence confirming lower relative absorbance change in them. Hence, the plasmonic optical effects of Au and Ag Mnps optimally influence the light harvesting ability of TiO_2_ at A3 fluence based photoanode.

### Photovoltaic characteristics of DSSCs

**Figure 11:**
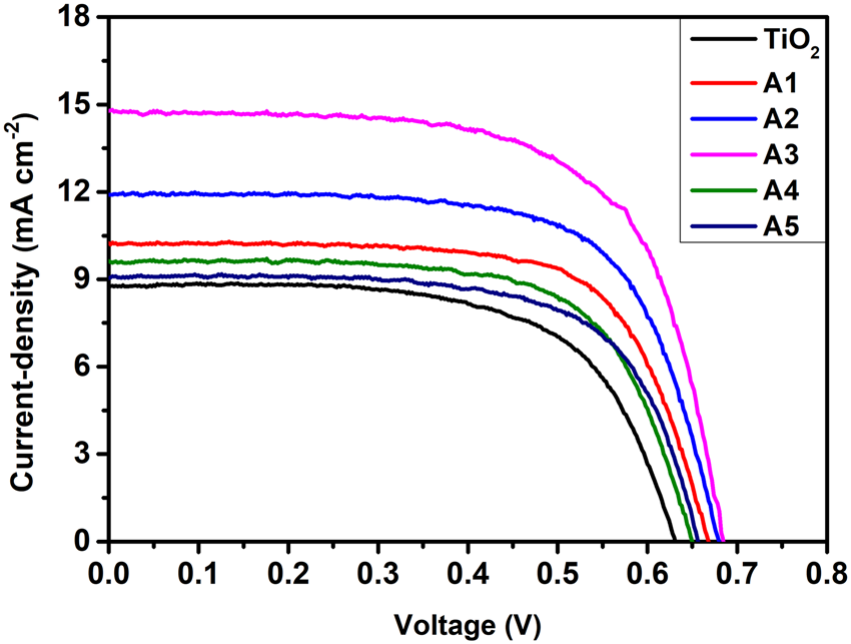
Device performances. Current-density – Voltage (J-V) curves of unimplanted and Au-Ag implanted TiO_2_ at varying fluence (A1–A5).

**Figure 12:**
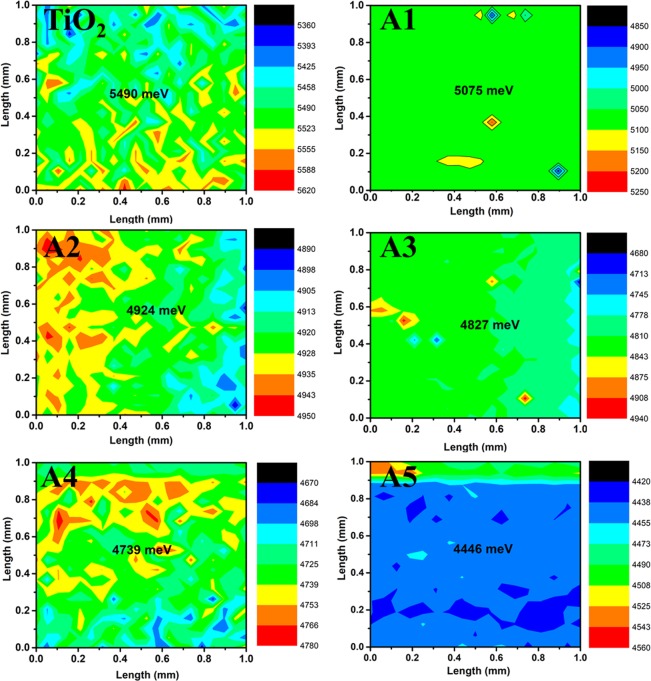
Work function measurements. Kelvin Probe work function images of unimplanted and Au-Ag implanted TiO_2_ at varying fluence (A1–A5).

**Figure 13:**
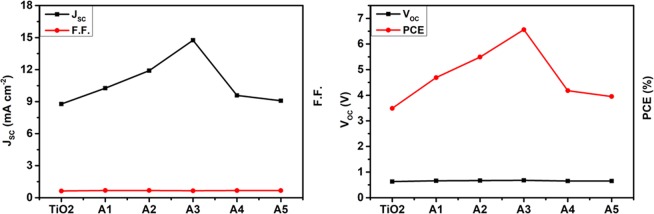
Variation in photovoltaic parameters. Variation of photo-voltaic parameters with different implantation fluence based photoanodes of DSSCs.

Unimplanted and Au-Ag implanted TiO_2_ films are used as photoanodes in the fabrication of DSSCs to test their photovoltaic performance under 1 sun illumination conditions (Intensity 100 mW cm^−2^). The photo-current density – voltage (J-V) curves of various DSSCs are shown in Fig. 11; and the obtained short circuit current-density (J_SC_), open circuit voltage (V_OC_), fill factor (F.F.) and PCE are tabulated in Table 2. PCE of Au-Ag implanted DSSCs are observed to be higher (A1 (4.69%), A2 (5.49%), A3 (6.56%), A4 (4.18%) and A5 (3.95%)) in comparison to unimplanted DSSC (3.49%); which is due to the enhanced J_SC_, V_OC_ as well as FF values. Momentous enhancement in J_SC_ of implanted DSSCs (A1 (10.26), A2 (11.90), A3 (14.75), A4 (9.59), and A5 (9.09) mA cm^−2^) relative to unimplanted DSSC (8.78 mA cm^−2^) originates not only from the enhanced light harvesting ability of TiO_2_, generating enormous amount of photo-excited electrons; but also from the plasmonic electrical effects induced by Au and Ag Mnps embedded in TiO_2_ photoanodes. The plasmonically excited Mnps also generate hot charge carriers *i.e*. electrons and fill the trap levels of TiO_2_, which helps in reducing the charge extraction barrier at C.B. of TiO_2_ and LUMO level of dye interface that stimulates the transfer of photo-generated electrons to the C.B. of TiO_2_; hence contributes to the enhanced J_SC_ values in plasmonic DSSCs. Furthermore, Au and Ag Mnps accumulate photo-generated electrons from dye molecules that eventually leads to the upward shifting of Fermi energy level (E_F_) of TiO_2_; and has been investigated from Kelvin-Probe work function ($\varphi$) measurements shown in Fig. 12. It has been observed that $\varphi$ values decreases in Au-Ag implanted TiO_2_ relative to unimplanted TiO_2_. Since, $\varphi$ is defined as the difference between vacuum energy (E_V_) and E_F_ level; and E_V_ is constant, E_F_ shifts towards C.B. of TiO_2_ photoanode; which, further is an indication of increased V_OC_ of implanted TiO_2_ based DSSCs (A1 (0.66 V), A2 (0.67 V), A3 (0.68 V), A4 (0.65 V) and A4 (0.65 V)) than unimplanted DSSC (0.63 V), as V_OC_ is the difference of E_F_ and redox potential of electrolyte; resulting in higher F.F. values. Moreover, it has been observed that PCE, J_SC_, V_OC_ and F.F. suddenly decreases at A4 and A5 based DSSC; due to the combined effect of lesser dye loading, generating lesser photo-generated charge carriers as well as higher recombination rate as Au and Ag start acting as charge recombination centers, preventing their transportation to TiO_2_ C.B. The variations in the photovoltaic parameters of the fabricated DSSCs are presented in Fig. 13. Hence, A3 based DSSC exhibited highest PCE with an augmentation of 87.97% in comparison to the unimplanted DSSCs.

**Table 2:** Photovoltaic and Work function parameters.

Sample Name	J_SC_ (mA cm^−2^)	V_OC_ (V)	PCE (%)	F.F.	*φ* (eV)
TiO_2_	8.78	0.63	3.49	0.63	5.49
A1	10.26	0.66	4.69	0.68	5.07
A2	11.90	0.67	5.49	0.68	4.92
A3	14.75	0.68	6.56	0.65	4.82
A4	9.59	0.65	4.18	0.67	4.73
A5	9.09	0.65	3.95	0.67	4.46

Photovoltaic and work function parameters of unimplanted and Au-Ag implanted TiO_2_ photoanodes at varying fluence based DSSCs.

**Figure 14:**
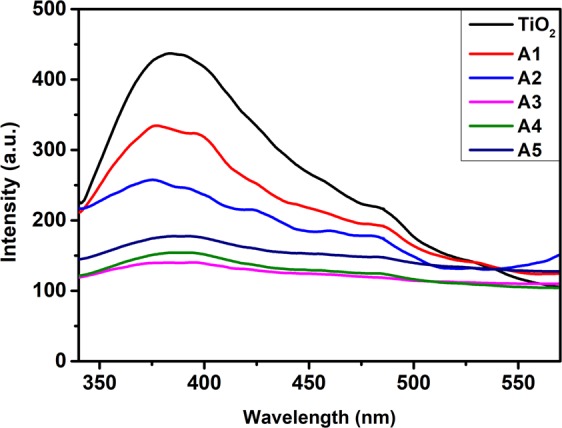
Emission spectra. PL emission spectra of unimplanted and Au-Ag implanted TiO_2_ at varying fluence (A1–A5).

The increased J_SC_ and V_OC_ *via* upward shifting of E_F_ level due to photo-generated electrons accumulation at Mnps supports the reduction in recombination rate of photo-generated charge carriers in Au-Ag implanted TiO_2_ photoanodes and is demonstrated by recording PL emission spectra. Since, PL emission arises due to the photons emitted during the recombination of photo-generated electron-hole pairs; offering lesser recombination rate for lower PL intensity. Figure 14 shows broad emissions around 330–550 nm in all the photoanodes; attributable to transitions involving singly ionized oxygen vacancies present in TiO_2_. A significant decrease is observed in PL intensity of Au-Ag implanted photoanodes; which further decreases with increased fluence up to A3 photoanodes; suggesting effective suppression of recombination rate, as now the photo-generated electrons gets stored at Au and Ag Mnps and hence prolongs the electron lifetime at photoanode, which is later confirmed through EIS measurements. A slight increase at A4 and A5 photoanodes arises due to the agglomeration of Au and Ag in TiO_2_ letting them act as charge recombination centers.

**Figure 15:**
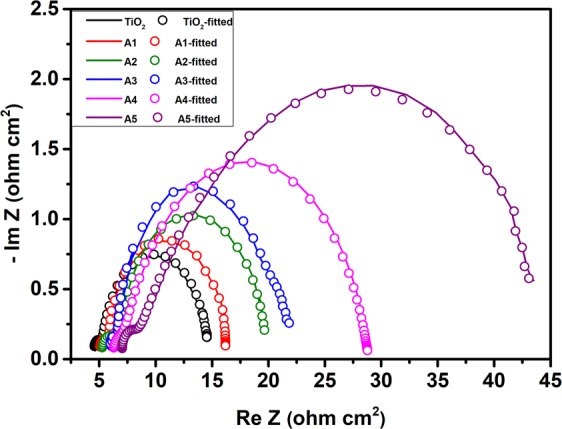
EIS characteristics. Nyquist plots obtained for unimplanted TiO_2_ and Au-Ag implanted TiO_2_ based DSSCs at different fluence (A1-A5) and fitted with equivalent circuit model ${R}_{s}+C/[{R}_{1}+Q/{R}_{2}]$.

Figure 15 depicts the Nyquist plots fitted with equivalent circuit model, measured for the unimplanted and Au-Ag implanted DSSCs; to understand their interfacial charge transfer mechanism. It exhibited two semicircles; at higher frequency fitted to resistance offered by reduction reactions at C.E./electrolyte interface (R_1_); and at intermediate frequency fitted to the charge transfer process at photoanode/electrolyte interface given by charge transfer resistance (R_2_) and charge transfer capacitance (*C*) along with a constant phase element (Q). The intercept along X-axis of high frequency semicircle represents equivalent series resistance (R_S_) having the contribution of FTO, unimplanted and Au-Ag implanted TiO_2_, Pt CE, and electrolyte of DSSCs; which slightly increases with increasing fluence of Au-Ag Mnps in TiO_2_ owing to the additional resistance induced by Au and Ag Mnps relative to unimplanted DSSC. R_1_ is observed to be analogous in all the fabricated DSSCs; because of the usage of same type of Pt CE in them. Substantial increase in R_2_ values is noticed with the embedment of Au-Ag in TiO_2_ based DSSCs; with continuous increment for increasing fluence from A1 to A5. It is referred to the hot carrier generation and charge storage ability of Au and Ag Mnps; that lead to decrease in the recombination rate of photo-generated charge carriers; resulting in increasing the electron lifetime at photoanode as well as its transport path length, which leads to efficient charge transportation in DSSCs.

**Figure 16:**
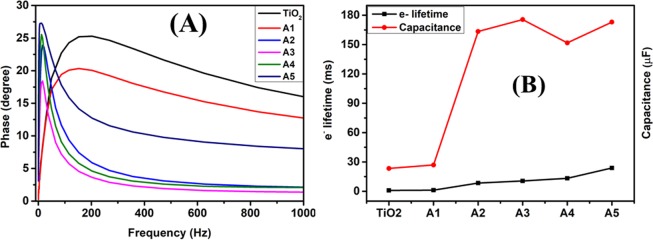
Bode plots, electron lifetime and capacitance calculations. (**A)** Bode plots obtained for unimplanted TiO_2_ and Au-Ag implanted TiO_2_ based DSSCs at varying fluence (A1-A5) and **(B)** Variation of electron lifetime (τ) and capacitance (C) with different photoanodes based DSSCs.

The electron lifetime calculations at TiO_2_ for all the fabricated DSSCs are investigated through Bode plots (Fig. 16(A)). Fabricated DSSCs exhibited a characteristic frequency maxima, indicating the transient processes occurring at photoanode/electrolyte interface, which shifts towards the lower frequency region with increasing Au-Ag embedment in TiO_2_; resulting in longer electron lifetime ($\tau$) calculated from the Eq. (3);

3 $$\tau =\frac{1}{2\pi {f}_{max}}$$

where, *f*_*max*_ is the frequency maxima corresponding to phase shift peak in Bode plots. Moreover, the elongated electron lifetime at photoanode further confirmed the increase in their transport path length. Further, the value of capacitance (*C*) at C.B. of TiO_2_ are measured from the expression, $C=\frac{\tau }{{R}_{2}}$; which indicates the increased charge storage ability of Au-Ag implanted DSSCs and this capacity enhances with increased fluence of Au-Ag Mnps, as the photo-generated electrons get accumulated on them. Figure 16(B) shows the variation of *τ* and *C* with the different fluence of Au-Ag implantation on TiO_2_ based DSSCs. The EIS parameters such as R_S_, R_2_, *C*, and *τ* for all the fabricated DSSCs are tabulated in Table 3. Although, Au-Ag embedment in TiO_2_ facilitates the charge transportation in DSSCs *via* reducing the recombination, enhancing the electron lifetime as well as charge storage ability; even at highest fluence (A5 based DSSC), still its PCE value is lower which confirmed the sole effect of lower dye loading.

**Table 3:** EIS parameters. R_S_, R_2_, *C*, and *τ* parameters of unimplanted and Au-Ag implanted TiO_2_ photoanodes at varying fluence based DSSCs.

Sample Name	R_S_ (Ω cm^2^)	R_2_ (Ω cm^2^)	*τ* (ms)	*C* (μF)
TiO_2_	4.45	9.3	0.87	23.38
A1	4.93	10.41	1.12	26.89
A2	5.29	12.91	8.44	163.43
A3	6.00	15.08	10.59	175.56
A4	6.36	21.83	13.25	151.74
A5	7.07	34.54	23.90	172.98

**Figure 17:**
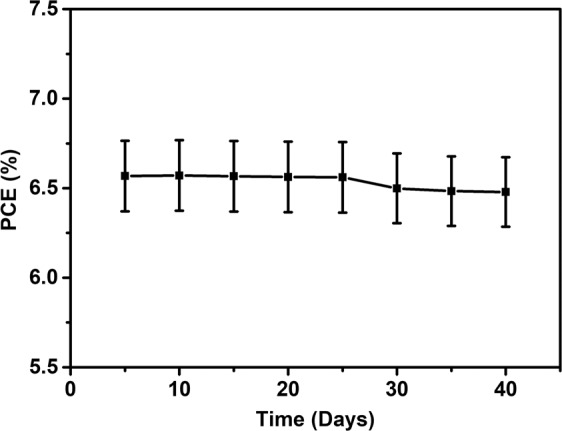
Long term stability test. Stability of PCE of Au-Ag implanted DSSC (Fluence 6 × 10^15^ ions cm^−2^) under 1 sun illumination (Intensity 100 mW cm^−2^).

Furthermore, A3 based DSSC exhibiting highest PCE, has been tested for photo-voltaic performance after an interval of 5 days for overall 40 days. Figure 17 shows a slight decrease in the PCE of A3 DSSC within initial days and then becomes negligibly constant; which indicates the long-term stability of Au and Ag Mnps in TiO_2_ protected with thin TiO_2_ melt from corrosion; hence representing long run durability of Au-Ag implanted DSSC.

Hence, optimized fluence representing maximum PCE enhancement of highly stable DSSC by 87.97%, due to balanced synergistic interactions involving plasmonic optical and electrical effects of Au and Ag Mnps, is 6 × 10^15^ ions cm^−2^.

## Conclusions

In conclusion, we were able to successfully optimize the bimetallic Au-Ag implantation in TiO_2_ photoanodes for the fabrication of highly efficient third generation DSSCs. Significant enhancement of 87.97% in PCE was achieved for Au-Ag implanted DSSC (Fluence- 6 × 10^15^ ions cm^−2^) with J_SC_ and V_OC_ of 14.75 mA cm^−2^ and 0.68 V, respectively in comparison to unimplanted DSSC (J_SC_ = 8.78 mA cm^−2^, V_OC_ = 0.63 V and PCE = 3.49%). It is attributed to (i) the enhanced and broadened absorbance of N719 dye sensitizer *via* simultaneous LSPR property of Au and Ag Mnps along with the hot carrier generation, which improves the light harvesting ability of TiO_2_ photoanodes; (ii) the reduced recombination reaction rate as Au and Ag Mnps in TiO_2_ exhibits charge storage ability, leading to increased charge transfer resistances at photoanode/electrolyte interface; (iii) improved interfacial charge carrier transfer processes *via* increased charge storage capacitance as well as photo-generated electron lifetime.

## Materials and Methods

### Materials procurement

Materials used for the fabrication of DSSCs such as Fluorine doped tin oxide (FTO), absolute ethanol, zinc powder, hydrochloric acid (HCl), titanium (IV) isopropoxide (TTIP), platinum (Pt) paste and Di-tetrabutylammonium *cis*-bis(isothiocyanato)bis(2,2′-bipyridyl-4,4′-dicarboxylato)ruthenium(II) (N719) dye with analytical grade quality were purchased from Sigma Aldrich. Titanium dioxide (TiO_2_) paste and redox electrolyte (iodide-tri iodide ${{\rm{I}}}^{-}/{{\rm{I}}}_{3}^{-}$ in 3-methoxypropionitrile (EL-HSE)) were procured from Dyesol, Australia.

### Ion Implantation on TiO_2_ and DSSCs fabrication

Patterned and pre-cleaned FTO substrates, were spin coated with TTIP solution forming a compact layer and were annealed at 450 °C for 30 min. TTIP solution was prepared by adding a solution mixture of 5 ml ethanol and 200 μl HCl into 5 ml ethanol; kept under continuous stirring for 3 hours, along with the drop wise addition of 50 μl TTIP. Further, TiO_2_ paste was doctor bladed onto the compact layer deposited FTO with effective thickness and area of 3 μm and 0.25 cm^2^, respectively; followed by thermal annealing at 450 °C for 30 min. The prepared TiO_2_ films were implanted firstly with Ag ion beam (Energy-120 KeV) at room temperature under high vacuum conditions using low energy negative ion implanter (Inter University Accelerator Centre (IUAC), New Delhi, India) at different fluence of 10^15^–10^16^ ions cm^−2^. Secondly, Au ion beam was implanted with energy 80 KeV onto Ag implanted TiO_2_ at respective similar fluence. The Au-Ag implanted TiO_2_ films are named as A1, A2, A3, A4 and A5 for 1 × 10^15^, 3 × 10^15^, 6 × 10^15^, 9 × 10^15^ and 1.2×10^16^ ions cm^−2^, respectively. Au and Ag penetrated up to 22 and 17 nm depth in TiO_2_ and are calculated using the stopping and range of ions in matter (SRIM) software. The unimplanted and Au-Ag implanted TiO_2_ were immersed into N719 dye (0.3 mM concentration) for 24 h. Pt was deposited on pre-cleaned FTO substrates and were annealed at 450 °C for 30 min. for the preparation of counter electrode (CE). DSSCs were assembled by sandwiching different photoanodes and Pt CE using redox electrolyte in them as an intermediate to complete the circuit.

### Characterizations

Crystal structure of unimplanted and Au-Ag implanted TiO_2_ films were determined with X-ray diffraction (XRD) using D8 FOCUS, Bruker Ettlingen with Cu K_α_ radiation (*λ* = 1.5418 Å, Current = 30 mA and Voltage = 40 kV) from 5–80° Bragg’s angle. X-ray photoelectron spectroscopy (XPS) was done for elemental analysis *via* MAC2 electron analyzer system interconnected with MBE machine (EVA-32 Riber, France) at an excitation energy of 1253.3 eV using Mg K_α_ X-ray beam; within binding energy (B.E.) range 10–1500 eV. System used was initially calibrated using Au 4f_7/2_ line with 84.0 eV B.E. Surface morphology of the prepared films were investigated using field emission scanning electron microscope (FESEM-Carl Zeiss, Supra 55). UV-Vis measurements were done using SHIMADZU, UV-VIS NIR 3600 spectrometer within 250–800 nm wavelength range. To perform the desorption experiments, the N719 dye was desorbed from unimplanted and Au-Ag implanted TiO_2_ using 0.1 M aqueous solution of potassium hydroxide (KOH) and further, the absorbance spectra of deloaded dye solutions were recorded. Photovoltaic parameters of fabricated DSSCs were studied using Keithley source meter (Model 2400) under 1 sun illumination at 1.5 G AM of intensity 100 mW cm^−2^ with OAI, TriSOL solar simulator; which was initially calibrated with standard silicon cell prior to the measurements. The work function measurements of unimplanted and Au-Ag implanted TiO_2_ were recorded using Kelvin Probe technique (SKP, Kelvin Probe 4.5). The photoluminescence (PL) spectra was obtained at an excitation wavelength of 310 nm using PerkinElmer, LS 55 Fluorescence Spectrometer. Electrochemical impedance spectroscopy (EIS) was performed using frequency response analyzer (FRA) connected with Autolab potentiostat/galvanostat (PGSTAT12) within the frequency range of 0.1 Hz to 1.0 MHz^19,20^.
